# The impact of sleep, physical activity and sedentary behaviour on symptoms of depression and anxiety before and during the COVID-19 pandemic in a sample of South African participants

**DOI:** 10.1038/s41598-021-02021-8

**Published:** 2021-12-15

**Authors:** R. Lewis, L. C. Roden, K. Scheuermaier, F. X. Gomez-Olive, D. E. Rae, S. Iacovides, A. Bentley, J. P. Davy, C. J. Christie, S. Zschernack, J. Roche, G. Lipinska

**Affiliations:** 1 UCT Sleep Sciences and Applied Cognitive Science and Experimental Neuropsychology Team (ACSENT), Department of Psychology, University of Cape Town, Cape Town, South Africa; 2 Department of Family Medicine, Faculty of Health Sciences, University of the Witwatersrand, Johannesburg, South Africa; 3 Department of Human Kinetics and Ergonomics, Rhodes University, Grahamstown, South Africa; 4 MRC/Wits Rural Public Health and Health Transitions Research Unit (Agincourt), School of Public Health, Faculty of Health Sciences, University of the Witwatersrand, Johannesburg, South Africa; 5 Brain Function Research Group, School of Physiology, Faculty of Health Sciences, University of the Witwatersrand, Johannesburg, South Africa; 6 Division of Exercise Science and Sports Medicine, Department of Human Biology, Faculty of Health Sciences, University of Cape Town, Cape Town, South Africa; 7 Faculty Research Centre for Sport, Exercise and Life Sciences, School of Life Sciences, Faculty of Health and Life Sciences, Coventry University, Coventry, CV1 2DS UK

**Keywords:** Psychology, Health care

## Abstract

During lockdowns associated with the COVID-19 pandemic, individuals have experienced poor sleep quality and sleep regularity, changes in lifestyle behaviours, and heightened depression and anxiety. However, the inter-relationship and relative strength of those behaviours on mental health outcomes is still unknown. We collected data between 12 May and 15 June 2020 from 1048 South African adults (age: 32.76 ± 14.43 years; *n* = 767 female; *n* = 473 students) using an online questionnaire. Using structural equation modelling, we investigated how insomnia symptoms, sleep regularity, exercise intensity/frequency and sitting/screen-use (sedentary screen-use) interacted to predict depressive and anxiety-related symptoms before and during lockdown. We also controlled for the effects of sex and student status. Irrespective of lockdown, (a) more severe symptoms of insomnia and greater sedentary screen-use predicted greater symptoms of depression and anxiety and (b) the effects of sedentary screen-use on mental health outcomes were mediated by insomnia. The effects of physical activity on mental health outcomes, however, were only significant during lockdown. Low physical activity predicted greater insomnia symptom severity, which in turn predicted increased depressive and anxiety-related symptoms. Overall, relationships between the study variables and mental health outcomes were amplified during lockdown. The findings highlight the importance of maintaining physical activity and reducing sedentary screen-use to promote better sleep and mental health.

## Introduction

Insomnia and an irregular sleep pattern are intimately associated with symptoms of depression and anxiety^1,2^. Furthermore, lifestyle behaviours such as exercise and sedentary behaviour, influence these mental health outcomes independently of sleep quality^3–7^. During the COVID-19 pandemic, many sleep-related and lifestyle behaviours changed due to ‘lockdowns’ of populations which were introduced as one of the non-pharmaceutical interventions to manage the transmission of the SARS-CoV-2 virus^8–11^. During this period, increases in depressive and anxiety-related symptoms were also widely reported^12,13^. However, the inter-relationship between sleep and lifestyle factors regarding their strength and relative contribution to mental health outcomes during lockdown, has not been sufficiently explored. The pandemic-engendered lockdowns provide a unique opportunity to draw conclusions about the relative importance of these factors, which can then be applied clinically both in ordinary times and in times of significant disruption and stress.

Many studies show that sleep health is critical for mental health^14,15^. While there is strong, long-standing and undisputed evidence that insomnia is associated with both depression and anxiety^16,17^, more recent findings show that sleep regularity (the consistency in the times at which individuals are asleep and awake on a day-to-day basis) is also a contributor to these mental health outcomes^18,19^. In a sample of older adults, aged 45–84 years with diverse ethnic origins, and after controlling for age, sex and race, those with higher sleep irregularity presented with significantly higher clinical depressive symptoms^20^. The association between sleep regularity and anxiety is less clear, but several papers show that shift workers with irregular sleep patterns are likely to have an increased prevalence of anxiety^21,22^.

During lockdown, sleep disruption, primarily related to insomnia and poor global sleep quality, was associated with increased severity of depressive and anxiety-related symptoms^9,23–25^. However, it is unknown whether during the lockdown period (a) disturbances in sleep regularity were associated with negative mental health outcomes and (b) whether the association between sleep disturbance and mental health outcomes was more pronounced than during ordinary times.

Additionally, physical activity is important for managing symptoms of depression and anxiety^26–28^. The therapeutic benefits of exercise in managing mental health are most prominent when used in combination with standard treatment, although it can also be efficacious as a stand-alone therapy, albeit with smaller effect sizes^29,30^. During the COVID-19 lockdown period, Zhang et al.^31^ found that either too little or too much physical activity worsened negative emotions including depression and anxiety. They elaborated that, consistent with the U-shaped distribution of the relationship between negative emotion and physical activity, optimal levels of exercise were associated with significantly fewer negative emotions. Another study found that individuals who were active before lockdown but became inactive during lockdown, had increased depressive symptoms, feelings of loneliness and stress and decreased positive mental health^32^.

High levels of sedentary behaviour (defined as any waking behaviour in a sitting or reclining position, with energy expenditure ≤ 1.5 metabolic equivalent of task^33^) are associated with increased symptoms of depression and anxiety^34,35^. Furthermore, increased screen-use, another sedentary behaviour, is associated with increased risk of depression^5^ and anxiety, although in this case the association appears to be weaker than for other sedentary behaviours such as sitting or watching television^34^. Indeed, Meyer et al.^32^ also found that individuals who spent more time on screens during lockdown were more likely to experience an increase in depressive symptoms and loneliness and an overall worsening in mental health. However, increases in sitting time during lockdown were not associated with poorer mental health outcomes. These findings suggest that from a range of sedentary behaviours, increased screen-use is likely to be associated with detrimental mental health outcomes during lockdown, although it is unknown whether this effect is attributable to the sedentary nature of screen-use or some other factor, such as light exposure (particularly at night), or the content presented on screens (e.g., social media).

While each of these health and lifestyle factors is directly associated with depressive and anxiety-related outcomes, there is less research on the relationship between sleep and physical activity and their subsequent cumulative effects on mental health. One study in Brazil conducted during national lockdown, on a sample of adolescents, found that physical inactivity and high screen-use (television and computer use) were associated with worse mental health outcomes defined as increased feelings of loneliness, sadness and anxiety^36^. Furthermore, poor sleep quality partly mediated the association between variables of physical activity and sedentary behaviour (including screen-use) and mental health outcomes.

The lockdowns imposed during the COVID-19 pandemic allow for the investigation of multiple factors (their combinations and relative contributions) to describe some of the most important everyday determinants of mental health, under conditions of considerable stress. Our study aimed to use structural equation modelling to comprehensively examine the impact of physical activity, sedentary behaviour (including screen-use) and sleep health (including sleep regularity and insomnia severity symptoms) on symptoms of depression and anxiety before and during national lockdown in a sample of South Africans.

Based on the literature garnered from ordinary and pandemic life, we tested the following hypotheses:

1. Greater insomnia severity symptoms and irregular sleep patterns will be directly associated with greater depressive and anxiety-related symptoms and this association will be stronger during lockdown than before lockdown.
2. Reduced physical activity and increased sedentary behaviour (including screen-use), will be directly associated with more depressive and anxiety-related symptoms and this association will be stronger during lockdown than before lockdown.
3. Reduced physical activity and increased sedentary behaviour (including screen-use), will be indirectly associated with more depressive and anxiety-related symptoms via their influence on sleep health and this association will be stronger during lockdown than before lockdown.

## Results

### Participant characteristics

Descriptive data on all study variables are presented as mean ± standard deviation (SD) or median with interquartile range (IQR) in Table 1. A quarter (26.6%) of participants endorsed a previous clinical diagnosis of depression and a third (30.4%) reported a previous clinical diagnosis of anxiety.

**Table 1:** Descriptive characteristics of variables before and during lockdown.

	Before lockdown	During lockdown
ISI score	4 (0, 26)^f^	10 (0, 28)^f^
Bedtime regularity	2 (1, 7)^f^	4 (1, 7)^f^
Wake-up time regularity	1 (1, 7)^f^	3 (1, 7)^f^
Sleep duration regularity	2 (1, 7)^f^	4 (1, 7)^f^
MPA (min/day)	30 (0, 180)^a^	10 (0, 180)^b^
VPA (min/day)	30 (0, 180)^a^	15 (0, 180)^b^
Total screen time (min/day)	464 ± 208^b^	667 ± 228^c^
Sitting time (min/day)	390 ± 184^d^	554 ± 221^e^
PHQ-2 score	1 (0, 6) ^f^	2 (0, 6)^f^
GAD-7 score	4 (0, 21)^f^	9 (0, 21)^f^

Data are presented as median (IQR) or mean (SD).

*ISI* Insomnia Severity Index, *MPA* moderate physical activity, *VPA* vigorous physical activity, *min* minutes, *PHQ-2* Patient Health Questionnaire-2, *GAD-7* General Anxiety Disorder-7.

^a^*N* = 1037.

^b^*N* = 1043.

^c^*N* = 1017.

^d^*N* = 1006.

^e^*N* = 1005.

^f^*N* = 1048.

### Model testing

#### Depressive symptoms

When assessed using the Before Lockdown dataset, goodness-of-fit indices of the hypothesised measurement model were within acceptable range (CFI = 0.983 and RMSEA = 0.034 [90% confidence interval (CI): 0.025–0.042]). To achieve these fit indices, we removed item 3 of the ISI from the model and allowed several items to covary (ISI items 1 and 2, ISI items 5 and 7, ISI items 2 and 4). Standardised factor loadings ranged between 0.404 and 0.983 (Supplementary Table S1). When fitting the model using the During Lockdown dataset, the measurement model achieved an acceptable fit (CFI = 0.965 and RMSEA = 0.056[90%CI 0.049–0.063]), with standardised factor loadings ranging from 0.534 to 0.872 (Supplementary Table S1).

**Figure 1:**
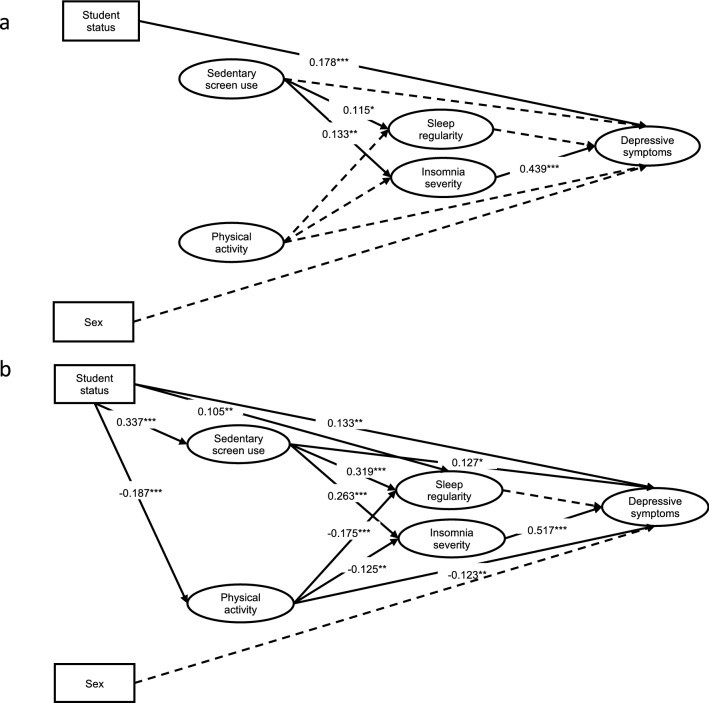
Structural equation model predicting depressive symptoms. This structural equation model predicts depressive symptoms from physical activity, sedentary screen-use, insomnia severity, and sleep regularity (with higher numbers indicating greater irregularity) before (**a**) and during (**b**) lockdown. Statistics are the observed standardized regression coefficients (measurement model excluded for readability). Solid lines represent significant paths. Dotted lines represent non-significant paths. All statistics conducted using *R* software^57^. *p < 0.05. **p < 0.01. ***p < 0.001.

Following the establishment of the measurement model, we estimated the relationships hypothesized between the observed and latent variables (see, Fig. 1 and Table 2). When tested on the Before Lockdown dataset, the structural model achieved acceptable fit (CFI = 0.950, RMSEA = 0.050 (90% CI 0.044–0.056). The model derived from the During Lockdown dataset had to be re-specified to account for the direct effect that student status has on (a) physical activity, sedentary screen-use, and sleep regularity, and (b) the covariation between item 2 on the ISI and sleep timing regularity. The final model fit the data relatively well (CFI = 0.950, RMSEA = 0.059 [90%CI 0.053–0.065]).

**Table 2:** Results of structural equation models predicting depressive symptoms (N = 1048).

Effects	Before lockdown				During lockdown			
	*β*	*p*	95% CI		*β*	*p*	95% CI	
			LL	UL			LL	UL
**Direct**								
Student status → Physical activity	–	–	–	–	− 0.187	0.001**	− 0.294	− 0.081
Student status → Screen-use	–	–	–	–	0.337	< 0.001***	0.257	0.417
Student status → Sleep regularity	–	–	–	–	0.105	0.002**	0.038	0.172
Physical activity → Sleep regularity	− 0.025	0.626	− 0.124	0.074	− 0.175	< 0.001***	− 0.265	− 0.085
Screen-use → Sleep regularity	0.115	0.030*	0.011	0.220	0.319	< 0.001***	0.230	0.408
Physical activity → Insomnia severity	0.036	0.414	− 0.051	0.123	− 0.125	0.009**	− 0.219	− 0.031
Sedentary screen-use → Insomnia severity	0.133	0.010**	0.032	0.234	0.263	< 0.001***	0.175	0.351
Physical activity → Depressive symptoms	0.018	0.678	− 0.066	0.102	− 0.123	0.007**	− 0.213	− 0.034
Screen-use → Depressive symptoms	0.076	0.104	− 0.016	0.168	0.127	0.011*	0.029	0.226
Sleep regularity → Depressive symptoms	0.001	0.989	− 0.074	0.075	0.054	0.305	− 0.049	0.157
Insomnia severity → Depressive symptoms	0.439	< 0.001***	0.346	0.532	0.517	< 0.001***	0.432	0.603
Sex → Depressive symptoms	− 0.018	0.573	− 0.079	0.044	− 0.054	0.095	− 0.117	0.009
Student status → Depressive symptoms	0.178	< 0.001***	0.103	0.253	0.113	0.002**	0.043	0.183
**Indirect**^**a**^								
Physical activity → Sleep regularity → Depressive symptoms	0.000	0.989	− 0.002	0.002	− 0.009	0.320	− 0.028	0.009
Screen-use → Sleep regularity → Depressive symptoms	0.000	0.989	− 0.009	0.009	0.017	0.309	− 0.016	0.050
Physical activity → Insomnia severity → Depressive symptoms	0.016	0.414	− 0.022	0.054	− 0.065	0.010*	− 0.114	− 0.015
Screen-use → Insomnia severity → Depressive symptoms	0.058	0.013*	0.012	0.104	0.136	< 0.001***	0.086	0.186
**Total**^**b**^								
Physical activity → Depressive symptoms	0.034	0.476	− 0.059	0.126	− 0.385	< 0.001***	− 0.525	− 0.244
Screen-use → Depressive symptoms	0.135	0.012*	0.030	0.239	0.617	< 0.001***	0.488	0.747
**Total effect**^**c**^	0.768	< 0.001***	0.567	0.970	0.968	< 0.001***	0.738	1.197

Sleep regularity: higher numbers indicate greater irregularity.

*Screen-use* sedentary screen-use, *β* standardized path coefficient, *CI* confidence interval, *LL* lower limit, *UL* upper limit.

*p < 0.05. **p < 0.01. ***p < 0.001.

^a^Indirect effect of physical activity and sedentary screen-use on depressive symptoms through sleep regularity or insomnia.

^b^Total effects of physical activity and sedentary screen-use, including direct and indirect effects, on depressive symptoms.

^c^The combined effect of all direct and indirect effects on depressive symptoms.

Results describing the contribution of the covariates suggest that, both before and during lockdown (a) students experienced greater symptoms of depression compared to non-students and (b) sex was not directly associated with depressive symptoms. When we re-specified the model built on the During Lockdown dataset to include the direct effects of student status on sleep and lifestyle variables, students reported poorer sleep regularity, greater sedentary screen-use and engaged in less physical activity compared to non-students.

The structural model confirmed that a direct relationship exists between insomnia severity and depressive symptoms both before and during lockdown. The direction of the coefficient indicates that more severe insomnia symptoms were associated with greater depressive symptoms and that this association was somewhat stronger during, rather than before, lockdown. The relationship between sleep regularity and depressive symptoms was non-significant both before and during lockdown.

Before lockdown, physical activity had no direct effect on sleep regularity, severity of insomnia symptoms, or depressive symptoms. During lockdown, however, less physical activity was directly associated with poorer sleep regularity, greater severity of insomnia, and greater depressive symptoms. Notably, during lockdown there was an indirect pathway from physical activity to depressive symptoms via insomnia severity.

Greater sedentary screen-use, both before and during lockdown, was associated with less sleep regularity, and greater severity of insomnia symptoms. The direct pathway between sedentary screen-use and depressive symptoms went from being non-significant before lockdown to significant during lockdown. Both before and during lockdown, an indirect pathway existed from sedentary screen-use to depressive symptoms via insomnia symptom severity. The directionality of the coefficients for the indirect pathway indicated that greater sedentary screen-use was associated with more severe insomnia symptoms and, therefore, more depressive symptoms. This association was stronger during lockdown than before lockdown.

#### Anxiety symptoms

When assessed using the Before Lockdown dataset, goodness-of-fit indices of the hypothesised measurement model were within acceptable range (CFI = 0.972, RMSEA = 0.040 [90%CI 0.034–0.045]). We achieved these fit indices by removing item 3 of the ISI from the model and allowing several items to covary (ISI items 1 and 2, ISI items 5 and 7, GAD-7 items 4 and 5). Standardised factor loadings ranged between 0.478 and 0.881 (Supplementary Table S2). When assessing the measurement model on the During Lockdown dataset, goodness-of-fit statistics indicated that the measurement model fitted the data well, with CFI = 0.967 and RMSEA = 0.049 (90%CI 0.044–0.054). Standardised factor loadings ranged between 0.531 and 0.902 (Supplementary Table S2).

**Figure 2:**
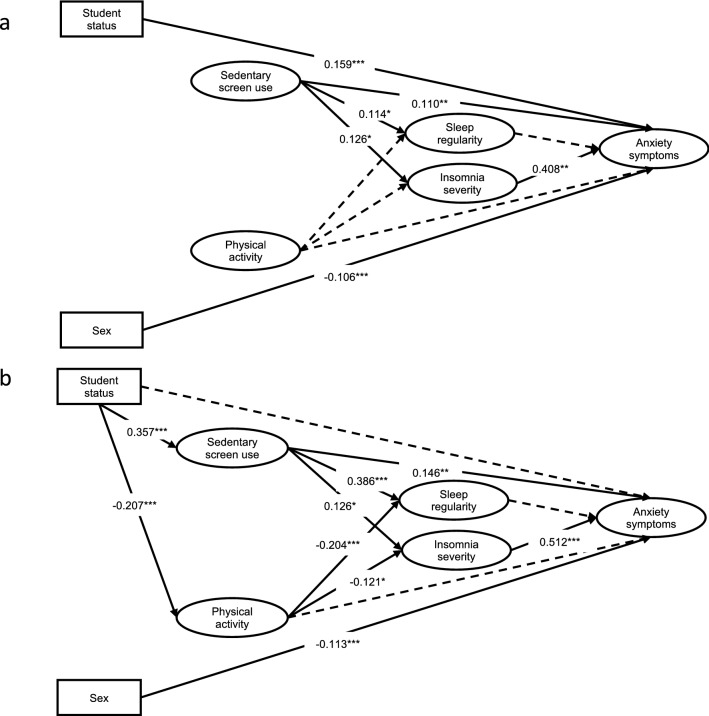
Structural equation model predicting anxiety symptoms. This structural equation model predicts anxiety symptoms from physical activity, sedentary screen-use, insomnia severity, and sleep regularity (with higher numbers indicating greater irregularity) before (**a**) and during (**b**) lockdown. Statistics are the observed standardized regression coefficients (measurement model excluded for readability). Solid lines represent significant paths. Dotted lines represent non-significant paths. All statistics conducted using *R* software^57^. *p < 0.05. **p < 0.01. ***p < 0.001.

Following the establishment of the measurement model, we estimated the structural relationships between the observed and latent variables (see Fig. 2 for a diagram of the structural results and Table 3 for regression paths results). When tested on the Before Lockdown dataset, the structural model achieved acceptable fit, with CFI = 0.951 and RMSEA = 0.048 (90%CI 0.043–0.052). When assessed using the During Lockdown dataset, we had to re-specify the structural model to account for the contribution of student status in order to show acceptable fit (CFI = 0.950, RMSEA = 0.054 [90%CI 0.050–0.059]). Specifically, student status was no longer directly associated with symptoms of anxiety, but rather influenced both sedentary screen-use and physical activity instead.

**Table 3:** Results of structural equation models predicting anxiety symptoms (N = 1048).

Effects	Before lockdown				During lockdown			
	*β*	*p*	95% CI		*β*	*p*	95% CI	
			LL	UL			LL	UL
**Direct**								
Student status → Physical activity	–	–	–	–	− 0.207	< 0.001***	− 0.312	− 0.081
Student status → Screen-use	–	–	–	–	0.357	< 0.001***	0.277	0.417
Physical activity → Sleep regularity	− 0.026	0.618	− 0.128	0.076	− 0.204	< 0.001***	− 0.300	− 0.109
Screen-use → Sleep regularity	0.114	0.030*	0.011	0.218	0.386	< 0.001***	0.299	0.473
Physical activity → Insomnia severity	0.034	0.455	− 0.055	0.123	− 0.121	0.015*	− 0.219	− 0.024
Screen-use → Insomnia severity	0.126	0.015*	0.024	0.228	0.259	< 0.001***	0.171	0.347
Physical activity → Anxiety symptoms	− 0.041	0.322	− 0.123	0.040	− 0.025	0.565	− 0.112	0.061
Screen-use → Anxiety symptoms	0.110	0.006**	0.032	0.188	0.146	0.002**	0.054	0.237
Sleep regularity → Anxiety symptoms	0.060	0.196	− 0.031	0.151	0.028	0.531	− 0.059	0.114
Insomnia severity → Anxiety symptoms	0.408	< 0.001***	0.329	0.486	0.512	< 0.001***	0.441	0.583
Sex → Anxiety symptoms	− 0.106	< 0.001***	− 0.160	− 0.052	− 0.113	< 0.001***	− 0.166	− 0.059
Student status → Anxiety symptoms	0.159	< 0.001***	0.098	0.220	0.006	0.857	− 0.056	0.068
**Indirect**^**a**^								
Physical activity → Sleep regularity → Anxiety symptoms	− 0.002	0.644	− 0.008	0.005	− 0.006	0.534	− 0.023	0.012
Screen-use → Sleep regularity → Anxiety symptoms	0.007	0.284	− 0.006	0.019	0.011	0.531	− 0.023	0.044
Physical activity → Insomnia severity → Anxiety symptoms	0.014	0.454	− 0.022	0.050	− 0.062	0.017*	− 0.113	− 0.011
Screen-use → Insomnia severity → Anxiety symptoms	0.051	0.020*	0.008	0.095	0.133	< 0.001***	0.085	0.180
**Total**^**b**^								
Physical activity → Anxiety symptoms	− 0.029	0.535	− 0.120	0.062	− 0.300	< 0.001***	− 0.432	− 0.168
Screen-use → Anxiety symptoms	0.168	< 0.001***	0.080	0.256	0.646	< 0.001***	0.518	0.774
**Total effect**^**c**^	0.660	< 0.001***	0.472	0.847	0.778	< 0.001***	0.572	0.985

Sleep regularity: higher numbers indicate greater irregularity.

*Screen-use* sedentary screen-use, *β* standardized path coefficient, *CI* confidence interval, *LL* lower limit, *UL* upper limit.

*p < 0.05, **p < 0.01. ***p < 0.0.

^a^Indirect effect of physical activity and sedentary screen-use on anxiety symptoms through sleep regularity or insomnia.

^b^Total effects of physical activity and sedentary screen-use, including direct and indirect effects, on anxiety symptoms.

^c^The combined effect of all direct and indirect effects on anxiety symptoms.

Results describing the contribution of the covariates suggest that both before and during lockdown males experienced less severe symptoms of anxiety compared to females. Additionally, before lockdown students experienced greater anxiety severity compared to non-students. However, during lockdown, this effect was no longer present. When we re-specified the model derived from the During Lockdown dataset to include the direct effects of student status on lifestyle variables, students engaged in greater sedentary screen-use and less physical activity compared to non-students.

The structural model confirmed that a direct relationship exists between insomnia symptom severity and anxiety symptoms both before lockdown and during lockdown. The direction of the coefficient indicated that more severe insomnia symptoms were associated with greater anxiety symptoms and that this association was somewhat stronger during lockdown than before lockdown. The relationship between sleep regularity and anxiety symptoms was non-significant both before and during lockdown.

Furthermore, the results showed that before lockdown, physical activity had no direct effect on sleep regularity, or severity of insomnia symptoms. During lockdown, however, less physical activity was directly associated with less sleep regularity and more severe insomnia symptoms. With regards to the effects of physical exercise on symptoms of anxiety, no relationship existed before lockdown. In addition, a significant indirect effect existed between physical activity and symptoms of anxiety during lockdown, such that less physical activity was associated with more severe insomnia symptoms, and thereby, greater anxiety symptoms.

Greater sedentary screen-use, both before and during lockdown, was significantly associated with poorer sleep regularity, increased severity of insomnia symptoms, and greater presence of anxiety symptoms. The associations between sedentary screen-use and sleep regularity was greater during lockdown than before lockdown. Moreover, both before and during lockdown there was an indirect pathway from sedentary screen-use to anxiety symptoms via insomnia symptom severity.

## Discussion

We investigated the contribution of multiple lifestyle and sleep health factors on symptoms of depression and anxiety in a sample of South African participants before and during lockdown Alert Level 5, using structural equation modelling. Additionally, on each respective model we controlled for the effects of sex, student status and previous clinical diagnoses of depression and anxiety. We aimed to determine how combinations of lifestyle and sleep-related factors influenced mental health outcomes during the lockdown instigated in response to the global COVID-19 pandemic.

We found that, consistent with our prediction and the literature^37,38^, greater insomnia symptom severity was associated with greater depressive and anxiety-related symptoms, irrespective of whether participants were in lockdown or not. The relationship between insomnia symptom severity and mental health outcomes, however, was somewhat greater during lockdown than before lockdown, and with similar increases for both symptoms of depression and anxiety. Few studies have examined participants’ sleep quality, duration or timing and its impact on mental health outcomes both before and during lockdown. Robillard et al.^39^ found that participants who had reduced time in bed or went to bed later (in comparison to those with extended time-in-bed), during lockdown than before lockdown, reported higher levels of stress, depression and anxiety.

In our sample, the relationship between insomnia symptom severity and mental health outcomes was the strongest amongst all the mental health and sleep/lifestyle associations. However, there was no association between sleep regularity and mental health outcomes at any time point. These results suggest that for symptoms of depression and anxiety (irrespective of the context), difficulties with initiating and maintaining sleep outweigh difficulties in maintaining a regular sleep schedule.

At odds with our prediction, modelling found no influence of time spent in moderate or vigorous physical activity on mental health outcomes before lockdown. This contrasts to previous literature and could be explained by the fact that individuals may make use of a variety of different tools to maintain a healthy mood and alleviate anxiety during ordinary life, such as socialising, entertainment and indoor and outdoor activities^40^. Amongst these, our data suggest that exercise is not a prominent coping tool under usual circumstances. However, during lockdown undertaking moderate or vigorous exercise was directly associated with a reduction in depressive symptoms, but not anxiety. Exercise may have a mood enhancing effect via its modulation of serotonergic and adrenergic activity in promoting brain-derived neurotrophic factor in the brain^41^. However, this exercise-related pharmacological action is not established in human or animal studies of anxiety.

Having high levels of sedentary behaviour, including screen-use, was associated with increased symptoms of depression and anxiety irrespective of the lockdown context. Similar associations have been found before lockdown, for example, in a study of Iranian children and adolescents investigating the relationship between screen time, physical activity and psychiatric distress^42^ and during lockdown in diverse contexts^43,44^. Moreover, a study of adolescents in China during the current COVID-19 pandemic suggests that engaging in physical activity and reducing sitting time were important to reduce symptoms of insomnia, depression and anxiety^45^.

Most notably several indirect relationships became relevant in predicting the burden of depressive and anxiety-related symptoms during the lockdown period. For example, individuals who exercised moderately or vigorously were less likely to report symptoms of insomnia and this lack of insomnia symptoms was associated with fewer depressive and anxiety-related symptoms. This effect was not present before lockdown, where there were presumably (a) less financial, household and childcare demands on many individuals, (b) a wide availability of a variety of outlets for stress, and (c) absent psychological impacts of quarantine that would have been present during the period of lockdown^46^. Importantly, although the proportion of individuals who exercised did decrease during the lockdown period (from 68.2% to 61.0%), more than half of the sample continued to exercise at home.

A similar pattern of findings was evident for sedentary behaviour. During the lockdown period (although also before lockdown), individuals who spent more time sitting and using screens were more likely to have poor sleep quality and in turn more severe symptoms of depression and anxiety. In summary, these findings bolster the tenet that during times of restriction and social isolation it is critical to be active and concurrently minimise sitting time and screen-use to maintain healthy sleep, and in turn, psychological well-being.

Our analyses also controlled for the effects of sex and student status. We found that being female was associated with higher symptoms of anxiety, independent of lockdown, but not depression. This latter finding is in contrast to most of the literature^47–49^ and may represent a unique resilient characteristic of females in our cohort. Other studies examining sex differences on the severity of depressive symptoms during lockdown generally follow the extant literature (females report higher levels of depressive symptoms than males). However, there are specific studies which report contrasting findings. For example, a recent longitudinal study compared, amongst a number of variables, depressive symptomatology at 3-weeks and 7-weeks into a lockdown period^50^. The authors found that at 3-weeks females had more severe depressive symptoms than males. However, at 7-weeks their symptoms had decreased somewhat. In contrast, their male counterparts had increased depressive symptoms at 7-weeks, with no between-sex difference in this mental health outcome at this timepoint. The authors concluded that females had greater long-term resilience to lockdown quarantine than males, although it is unclear to what extent this finding is generalizable from this cohort to other contexts, given the substantial variation in sex-specific changes in work and childcare arrangements globally.

Regarding student status, before lockdown being a student was associated with greater depressive and anxiety-related symptoms, consistent with prior literature^51^. This relationship remained during lockdown for depressive symptoms only. Most studies conducted during the pandemic have found that students, in contrast to non-students, experienced increased symptoms of both depression and anxiety^52^.

### Limitations and future directions

We note several important limitations. Firstly, our sampling method was not representative of the greater South African population and it likely underrepresents individuals living in urban low-income areas and rural districts.

Secondly, we note that our sample had a disproportionate number of female participants in comparison with male participants, which may have biased the results towards female participants in some analyses.

Thirdly, participants responses to questions asking about their experiences before lockdown may contain recall bias.

Fourthly, while our study described several relevant relationships between sleep/lifestyle variables and mental health outcomes, our findings are relatively general and cannot specify for whom these relationships are most salient. Future research should identify specific vulnerable and resilient individuals or groups by identifying how their sleep and lifestyle behaviours changed from ordinary life to the lockdown quarantine period.

## Conclusion

Many studies report on lifestyle, sleep and mental health outcomes during the COVID-19 pandemic. However, relatively few investigations have reported on the strength, directionality and relationships of multiple factors and examined these both before and during national lockdown. Our findings showed that, from a variety of sleep and lifestyle behaviours, difficulties with initiating and maintaining sleep had the most influence on symptoms of depression and anxiety, and that this relationship was exacerbated during lockdown in comparison with ordinary life. Furthermore, irrespective of the lockdown context, spending significant daily periods sitting and engaging on screens is detrimental for mental health. On the other hand, engaging in moderate to vigorous physical activity became especially relevant during lockdown with benefits to mental health. Our study shows that, in times of restriction and upheaval characteristic of lockdown, individuals can rely on physical activity as a valuable tool in managing and maintaining healthy sleep to support mental health. In the current context, where individuals are juggling multiple economic, social, educational and vocational factors, understanding which strategies are effective in reducing symptoms of depression and anxiety is an important priority.

## Methods

### Study design and setting

The data presented in this analysis are from a larger observational study examining multiple lifestyle, routine and mental health outcomes reported before and during COVID-19 Alert Level 5 lockdown in a sample of South African adults^10^. In this paper we focus on predicting symptoms of depression and anxiety based on two key lifestyle behaviours (physical activity and sedentary behaviour including screen-use [termed *sedentary screen-use*]) and two aspects of sleep health (insomnia severity and sleep regularity). We compare the estimated relationships between all of these factors before (defined as the three months prior to lockdown, i.e. January, February and March 2020) and during lockdown (defined as the five weeks of Alert Level 5 lockdown, i.e. 27 March to 30 April 2020). During Alert Level 5 lockdown in South Africa individuals were only allowed to leave the home to access essential services such as groceries and medical attention. Leaving the home to exercise was not permitted and the sale of alcohol and cigarettes was prohibited. Participants completed the online survey during Alert Levels 3 and 4 between 12 May and 15 June 2020.

### Participants and recruitment

We analysed data from 1048 South African adults (age: 32.76 ± 14.43 years; female: *n* = 767; male: *n* = 261; non-binary: *n* = 15; prefer not to say: *n* = 5). Of the sample, 473 were university students (age: 22.6 ± 6.39 years) and 575 were non-students (workers, retired individuals and volunteers; age: 41.1 ± 13.8 years). Volunteers completed an online survey in which they answered the same questions for two time points: before and during lockdown. The survey was distributed throughout South Africa using academic and social media networks together with snowball sampling techniques. We obtained ethical clearance from the Ethical Standards Committee at Rhodes University (2020-1459-3468) and the Department of Psychology’s Ethics Committee at the University of Cape Town (PSY2020-014). All methods were performed in accordance with the relevant guidelines and all participants gave informed consent prior to initiating the survey.

### Materials and instruments

The full range of demographic, routine, sleep, lifestyle and mental health variables collected in this survey have been reported elsewhere^10^. Here we focus on the variables relevant to our current hypotheses.

The Insomnia Severity Index (ISI)^53^ was used to characterise participants’ perceptions of their insomnia symptoms. The ISI comprises seven items that assess participants’ perceived difficulties with sleep initiation and maintenance, evaluation of their sleep quality (satisfaction and distress) and the daytime impact of their sleep difficulties. Scores range from 0 to 28 and the cut-off scores are specified as follows: 0–7 indicates no clinically significant insomnia; 8–14 shows subthreshold insomnia; 15–21 indicates clinical insomnia (moderate severity); and 22–28 indicates clinical insomnia (severe).

Participants reported their sleep regularity in terms of timing and sleep duration. Participants were asked to indicate the usual difference between their earliest and latest bedtimes and wake-up times (sleep timing regularity) and the usual difference between their longest and shortest sleep time (sleep duration regularity) in any given week before and during the lockdown period. Sleep timing regularity was calculated as the average self-reported bedtime and wake-up time regularity. Each question consisted of seven possible answers (< 1 h, 1 h, 1.5 h, 2 h, 2.5 h, 3 h, > 3 h variation in a given week), scored from 1 (< 1 h) to 7 (> 3 h), with higher scores indicating more irregularity.

Lifestyle factors included questions about exercise habits and screen-use duration (min/day). We used the International Physical Activity Questionnaire-short form (IPAQ)^54^ to describe minutes per day spent doing moderate physical activity (MPA) and vigorous physical activity (VPA) as well as time spent sitting (min/day).

We used the Patient Health Questionnaire-2 (PHQ-2)^55^, which consists of two items of the PHQ-2 to assess primary symptoms of depression (feeling down, depressed or hopeless; having little interest or pleasure in doing). Summed scores range from 0 to 6, with higher scores indicating greater levels of depression. Scores of 3 or more are suggestive of a depressive disorder. We used the Generalized Anxiety Disorder 7-item (GAD-7) scale^56^ to assess participants’ symptoms of anxiety. This questionnaire is designed to screen for generalised anxiety disorder. Scores range from 0–28, with higher scores indicating greater levels of anxiety. Scores of ≥ 5 indicate mild symptoms, ≥ 10 show moderate symptoms, while scores ≥ 15 are suggestive of severe symptoms of anxiety.

### Statistical analyses and structural equation modelling

All data analyses were completed using R statistical software and packages^57^. Direct comparisons of before-to-during lockdown variables including self-reported insomnia symptoms, sleep regularity, physical activity, sitting time, screen-use, depressive symptoms and anxiety-related symptoms have been reported before^10^. Those data also examined the contribution of sex (female versus male) and student status (non-student versus student) on sleep, lifestyle and mental health outcomes and found that before-to-during lockdown comparisons differed significantly by sex and student status. Hence, we controlled for sex and student status in our model testing analyses. Furthermore, we also controlled for the effects of previous diagnoses of depression and anxiety. Where these control variables did not alter the results, we excluded them from the final models.

We used a structural equation modelling (SEM) framework to estimate the hypothesised model using the “lavaan” package^58^. All continuous variables were scaled before being entered into the model. Four separate models explored depressive and anxiety-related symptoms before and during lockdown (Before Lockdown and During Lockdown datasets). SEM models were fitted using full information maximum likelihood estimation (FIML) to reduce bias due to missing data and maximum likelihood estimation with robust standard errors to ensure multivariate normality^59,60^.

First, we tested each of the four models to establish whether the latent variables are explained by the indicators using confirmatory factor analysis (CFA). The latent variables were insomnia severity (7 items of the ISI), sleep regularity (sleep timing regularity and sleep duration regularity), physical activity (time spent in moderate and vigorous exercise), sedentary screen-use (sitting time and total screen-use), depression (both items from the PHQ-2) and anxiety (7 items from the GAD-7). After establishing the measurement model, the structural relationships between the observed (e.g., sex and student status) and latent variables were estimated. Model fit was evaluated using the comparative fit index (CFI) and the root mean square error of approximation (RMSEA). An adequate fit is indicated by CFI > 0.95 and RMSEA ≤ 0.06^61^. If the hypothesised measurement model did not have a good fit, items whose factor loadings were ≤ 0.4 were dropped^62^. Furthermore, modification indices regarding correlated residual variances and covariances were included in the model if they fit theoretically^63^.

## Supplementary Information

Supplementary Information.
