# Supplementary material for: The impact of sleep, physical activity and sedentary behaviour on symptoms of depression and anxiety before and during the COVID-19 pandemic in a sample of South African participants
Source: Sci Rep. 2021 Dec 15;11:24059. doi: 10.1038/s41598-021-02021-8 (PMC8674220; doi:10.1038/s41598-021-02021-8)
Supplement: Supplementary file 1 — Supplementary Information. [file 41598_2021_2021_MOESM1_ESM.docx]

**SUPPLEMENTARY MATERIAL**

**Patient Health Questionnaire-2 (PHQ-2)**

Please choose which of the following four options best applies to you for each question BEFORE and DURING lockdown.

0 = Not at all

1 = Several days

2 = More than half of the days

3 = Nearly every day

| 1. Little interest or pleasure in doing things |  |  |  |  |
| --- | --- | --- | --- | --- |
| BEFORE lockdown | 0 | 1 | 2 | 3 |
| DURING lockdown | 0 | 1 | 2 | 3 |
| 2. Feeling down, depressed or hopeless |  |  |  |  |
| BEFORE lockdown | 0 | 1 | 2 | 3 |
| DURING lockdown | 0 | 1 | 2 | 3 |

#

# **Generalized Anxiety Disorder 7-item (GAD-7) scale**

Please choose which of the following four options best applies to you for each question BEFORE and DURING lockdown.

0 = Not at all

1 = Several days

2 = More than half of the days

3 = Nearly every day

| 1. Feeling nervous, anxious, or on edge |  |  |  |  |
| --- | --- | --- | --- | --- |
| BEFORE lockdown | 0 | 1 | 2 | 3 |
| DURING lockdown | 0 | 1 | 2 | 3 |
| 2. Not being able to stop or control worrying |  |  |  |  |
| BEFORE lockdown | 0 | 1 | 2 | 3 |
| DURING lockdown | 0 | 1 | 2 | 3 |
| 3. Worrying too much about different things |  |  |  |  |
| BEFORE lockdown | 0 | 1 | 2 | 3 |
| DURING lockdown | 0 | 1 | 2 | 3 |
| 4. Trouble relaxing |  |  |  |  |
| BEFORE lockdown | 0 | 1 | 2 | 3 |
| DURING lockdown | 0 | 1 | 2 | 3 |
| 5. Being so restless that it’s hard to sit still |  |  |  |  |
| BEFORE lockdown | 0 | 1 | 2 | 3 |
| DURING lockdown | 0 | 1 | 2 | 3 |
| 6. Becoming easily annoyed or irritable |  |  |  |  |
| BEFORE lockdown | 0 | 1 | 2 | 3 |
| DURING lockdown | 0 | 1 | 2 | 3 |
| 7. Feeling afraid as if something awful might happen |  |  |  |  |
| BEFORE lockdown | 0 | 1 | 2 | 3 |
| DURING lockdown | 0 | 1 | 2 | 3 |

# **Sleep Regularity Questions**

1 = Less than 1 hour

2 = 1 hour

3 = 1.5 hours

4 = 2 hours

5 = 2.5 hours

6 = 3 hours

7 = More than 3 hours

1. Bedtime regularity: What was the usual difference between your earliest and latest bedtime in any given week?

| BEFORE lockdown | 1 | 2 | 3 | 4 | 5 | 6 | 7 |
| --- | --- | --- | --- | --- | --- | --- | --- |
| DURING lockdown | 1 | 2 | 3 | 4 | 5 | 6 | 7 |

1. Wake-up time regularity: What was the usual difference between your earliest and latest wake-up time in any given week?

| BEFORE lockdown | 1 | 2 | 3 | 4 | 5 | 6 | 7 |
| --- | --- | --- | --- | --- | --- | --- | --- |
| DURING lockdown | 1 | 2 | 3 | 4 | 5 | 6 | 7 |

1. Sleep duration regularity: What was the usual difference between your shortest and longest sleep time in any given week?

| BEFORE lockdown | 1 | 2 | 3 | 4 | 5 | 6 | 7 |
| --- | --- | --- | --- | --- | --- | --- | --- |
| DURING lockdown | 1 | 2 | 3 | 4 | 5 | 6 | 7 |

**The Insomnia Severity Index (ISI)**

The next seven questions relate to insomnia-like problems that were present on most nights during two distinct 2-week periods: BEFORE lockdown: The two-week period prior to lockdown (i.e. mid-March 2020). DURING lockdown: The two-week period during level 5 lockdown that is most representative of your sleep problems

For each question, please rate the SEVERITY of your insomnia problem(s) by selecting the word that best describes your answer.

| 1. Difficulty falling asleep | None | Mild | Moderate | Severe | Very Severe |
| --- | --- | --- | --- | --- | --- |
| BEFORE lockdown | 0 | 1 | 2 | 3 | 4 |
| DURING lockdown | 0 | 1 | 2 | 3 | 4 |
| 2. Difficulty staying asleep | None | Mild | Moderate | Severe | Very Severe |
| BEFORE lockdown | 0 | 1 | 2 | 3 | 4 |
| DURING lockdown | 0 | 1 | 2 | 3 | 4 |
| 3. Problems waking up too early | None | Mild | Moderate | Severe | Very Severe |
| BEFORE lockdown | 0 | 1 | 2 | 3 | 4 |
| DURING lockdown | 0 | 1 | 2 | 3 | 4 |
| 4. How satisfied/dissatisfied were you with your sleep pattern? | Very satisfied | Satisfied | Neutral | Dissatisfied | Very dissatisfied |
| BEFORE lockdown | 0 | 1 | 2 | 3 | 4 |
| DURING lockdown | 0 | 1 | 2 | 3 | 4 |
| 5. How noticeable to others do you think your sleep problem was in terms of impairing the quality of your life? | Not at all | Noticeable a little | Somewhat | Much | Very much noticeable |
| BEFORE lockdown | 0 | 1 | 2 | 3 | 4 |
| DURING lockdown | 0 | 1 | 2 | 3 | 4 |
| 6. How worried/distressed were you about your sleep problems? | Not at all | Noticeable a little | Somewhat | Much | Very much noticeable |
| BEFORE lockdown | 0 | 1 | 2 | 3 | 4 |
| DURING lockdown | 0 | 1 | 2 | 3 | 4 |
| 7. To what extent did you consider your sleep problem to interfere with your daily functioning (e.g. daytime fatigue, mood, ability to function at work/daily chores, concentration, memory, mood, etc.)? | Not at all | Interfering a little | Somewhat | Much | Very much interfering |
| BEFORE lockdown | 0 | 1 | 2 | 3 | 4 |
| DURING lockdown | 0 | 1 | 2 | 3 | 4 |

#

# **Physical Activity**

Think about all the physical activity that you did during a typical week BEFORE and DURING lockdown.

Moderate activities refer to activities that take moderate physical effort and make you breathe somewhat harder than normal. Think only about those physical activities that you did for at least 10 minutes at a time.

Vigorous physical activities refer to activities that take hard physical effort and make you breathe much harder than normal. Think only about those physical activities that you did for at least 10 minutes at a time.

1. How much time did you usually spend doing moderate physical activities on one of those days (in minutes - if not applicable, type 0) BEFORE lockdown?
2. How much time did you usually spend doing moderate physical activities on one of those days (in minutes - if not applicable, type 0) DURING lockdown?
3. How much time did you usually spend doing vigorous physical activities on one of those days (in minutes - if not applicable, type 0) BEFORE lockdown?
4. How much time did you usually spend doing vigorous physical activities on one of those days (in minutes - if not applicable, type 0) DURING lockdown?

**Sedentary Screen-Use**

This question is about the time you spent sitting on weekdays during a typical week BEFORE and DURING lockdown.

Include time spent at work, at home, while doing course work and during leisure time. This may include time spent sitting at a desk, visiting friends, reading, or sitting or lying down to watch television.

1. How much time did you usually spend sitting on a weekday BEFORE lockdown?
2. How much time did you usually spend sitting on a weekday DURING lockdown?

This question is about the time you spent on screens during a typical day BEFORE and DURING lockdown.

Screens include: phone, tablet, laptop, computer, TV, gaming console

1. Approximately how many hours during the DAYTIME (i.e. between sunrise and sunset) did you spend on screens BEFORE lockdown?
2. Approximately how many hours during the DAYTIME (i.e. between sunrise and sunset) did you spend on screens DURING lockdown?
3. Approximately how many hours during the NIGHT TIME (i.e. between sunset and sunrise) did you spend on screens BEFORE lockdown?
4. Approximately how many hours during the NIGHT TIME (i.e. between sunset and sunrise) did you spend on screens DURING lockdown?

#

**Table S1.** *Results of Depression Measurement Model*

|  |  | Before Lockdown | | | | During Lockdown | | | |
| --- | --- | --- | --- | --- | --- | --- | --- | --- | --- |
|  |  |  |  | 95% CI | |  |  | 95% CI | |
| Factor | Item | CR | Standardised factor loading | LL | UL | CR | Standardised factor loading | LL | UL |
| Insomnia Symptom Severity |  | 0.86 |  |  |  | 0.89 |  |  |  |
|  | ISI item 1 |  | 0.591* | 0.534 | 0.649 |  | 0.724* | 0.688 | 0.759 |
|  | ISI item 2 |  | 0.460* | 0.389 | 0.531 |  | 0.534* | 0.479 | 0.589 |
|  | ISI item 4 |  | 0.758* | 0.725 | 0.790 |  | 0.806* | 0.782 | 0.830 |
|  | ISI item 5 |  | 0.732* | 0.677 | 0.788 |  | 0.770* | 0.732 | 0.807 |
|  | ISI item 6 |  | 0.884* | 0.859 | 0.909 |  | 0.872* | 0.851 | 0.893 |
|  | ISI item 7 |  | 0.824* | 0.787 | 0.862 |  | 0.861* | 0.836 | 0.885 |
| Sleep Regularity |  | 0.77 |  |  |  | 0.81 |  |  |  |
|  | Sleep timing regularity |  | 0.744* | 0.658 | 0.829 |  | 0.864* | 0.819 | 0.908 |
|  | Sleep duration regularity |  | 0.834* | 0.743 | 0.925 |  | 0.786* | 0.739 | 0.832 |
| Physical Activity |  | 0.56 |  |  |  | 0.52 |  |  |  |
|  | MPA (min/day) |  | 0.739* | 0.519 | 0.960 |  | 0.540* | 0.415 | 0.666 |
|  | VPA (min/day) |  | 0.495* | 0.342 | 0.649 |  | 0.648* | 0.523 | 0.774 |
| Sedentary Screen-Use |  | 0.64 |  |  |  | 0.63 |  |  |  |
|  | Total screen time (min/day) |  | 0.643* | 0.485 | 0.801 |  | 0.670* | 0.562 | 0.777 |
|  | Sitting time (min/day) |  | 0.728* | 0.552 | 0.905 |  | 0.691* | 0.578 | 0.803 |
| Depressive Symptoms |  | 0.69 |  |  |  | 0.71 |  |  |  |
|  | PHQ2 Item 1 |  | 0.404* | 0.311 | 0.498 |  | 0.655* | 0.591 | 0.719 |
|  | PHQ2 Item 2 |  | 0.983* | 0.822 | 1.143 |  | 0.820* | 0.768 | 0.873 |

*Note.* CR = composite reliability, CI = confidence interval; LL = lower limit; UL = upper limit, MPA = moderate intensity physical activity, VPA = vigorous intensity physical activity, min = minutes, ISI = Insomnia Severity Index, PHQ2 = Patient Health Questionnaire-2. Sleep timing regularity = (wake-up regularity + bedtime regularity) / 2. Despite the relatively poor composite reliability for Physical activity and Sedentary screen-use, the items provide the best available measure of the hypothesised latent variable and, since error variance is accounted for when using a structural equation model, we continued with our analysis.

**p* < .0001.

**Table S2.** *Results of Anxiety Measurement Model*

|  |  | Before Lockdown | | | | During Lockdown | | | |
| --- | --- | --- | --- | --- | --- | --- | --- | --- | --- |
|  |  |  |  | 95% CI | |  |  | 95% CI | |
| Factor | Item | CR | Standardised factor loading | LL | UL | CR | Standardised factor loading | LL | UL |
| Insomnia Symptom Severity |  | 0.87 |  |  |  | 0.90 |  |  |  |
|  | ISI item 1 |  | 0.590* | 0.532 | 0.647 |  | 0.726* | 0.691 | 0.762 |
|  | ISI item 2 |  | 0.478* | 0.409 | 0.547 |  | 0.555* | 0.503 | 0.607 |
|  | ISI item 4 |  | 0.763* | 0.731 | 0.794 |  | 0.811* | 0.788 | 0.835 |
|  | ISI item 5 |  | 0.735* | 0.681 | 0.789 |  | 0.767* | 0.729 | 0.804 |
|  | ISI item 6 |  | 0.881* | 0.855 | 0.906 |  | 0.873* | 0.852 | 0.894 |
|  | ISI item 7 |  | 0.824* | 0.787 | 0.861 |  | 0.854* | 0.829 | 0.880 |
| Sleep Regularity |  | 0.77 |  |  |  | 0.81 |  |  |  |
|  | Sleep timing regularity |  | 0.763* | 0.681 | 0.845 |  | 0.855* | 0.810 | 0.899 |
|  | Sleep duration regularity |  | 0.813* | 0.728 | 0.898 |  | 0.794* | 0.748 | 0.839 |
| Physical Activity |  | 0.55 |  |  |  | 0.52 |  |  |  |
|  | MPA (min/day) |  | 0.509* | 0.366 | 0.651 |  | 0.531* | 0.385 | 0.677 |
|  | VPA (min/day) |  | 0.720* | 0.527 | 0.913 |  | 0.659* | 0.504 | 0.815 |
| Sedentary Screen-Use |  | 0.65 |  |  |  | 0.63 |  |  |  |
|  | Total screen time (min/day) |  | 0.605* | 0.468 | 0.741 |  | 0.681* | 0.561 | 0.801 |
|  | Sitting time (min/day) |  | 0.774* | 0.607 | 0.940 |  | 0.679* | 0.558 | 0.800 |
| Anxiety Symptoms |  | 0.91 |  |  |  | 0.91 |  |  |  |
|  | GAD7 Item 1 |  | 0.852* | 0.825 | 0.878 |  | 0.853* | 0.832 | 0.874 |
|  | GAD7 Item 2 |  | 0.878* | 0.851 | 0.906 |  | 0.902* | 0.884 | 0.921 |
|  | GAD7 Item 3 |  | 0.830* | 0.803 | 0.857 |  | 0.888* | 0.871 | 0.905 |
|  | GAD7 Item 4 |  | 0.694* | 0.645 | 0.744 |  | 0.782* | 0.752 | 0.811 |
|  | GAD7 Item 5 |  | 0.528* | 0.461 | 0.594 |  | 0.602* | 0.556 | 0.649 |
|  | GAD7 Item 6 |  | 0.616* | 0.562 | 0.670 |  | 0.665* | 0.625 | 0.705 |
|  | GAD7 Item 7 |  | 0.685* | 0.633 | 0.737 |  | 0.712* | 0.675 | 0.749 |

*Note.* CR = composite reliability, CI = confidence interval; LL = lower limit; UL = upper limit, MPA = moderate intensity physical activity, VPA = vigorous intensity physical activity, min = minutes, ISI = Insomnia Severity Index, GAD7 = General Anxiety Disorder-7. Sleep timing regularity = (wake-up regularity + bedtime regularity) / 2. Despite the relatively poor composite reliability for physical activity and sedentary screen-use, the items provide the best available measure of the hypothesised latent variable and, since error variance is accounted for when using an SEM, we continued with our analysis.

**p* < .0001.
